# Chlorinated Anilines as Molecular Templates to Achieve [2 + 2] Cycloaddition Reactions within Organic Cocrystals

**DOI:** 10.1021/acsomega.5c01991

**Published:** 2025-05-21

**Authors:** Grace K. White, Daniel K. Unruh, Herman R. Krueger, Ryan H. Groeneman

**Affiliations:** † Department of Natural Sciences and Mathematics, 7549 Webster University, St. Louis, Missouri 63119, United States; ‡ Office of the Vice President for Research, 4083 University of Iowa, Iowa City, Iowa 52242, United States

## Abstract

The cocrystallization of either 2,3,5,6-tetrachloroaniline (C_6_H_3_Cl_4_N) or 2,4,6-trichloroaniline (C_6_H_4_Cl_3_N) with *trans*-1,2-bis­(4-pyridyl)­ethylene (BPE) results in a pair of three-component hydrogen-bonded cocrystals, namely 2­(C_6_H_3_Cl_4_N)·(BPE) and 2­(C_6_H_4_Cl_3_N)·(BPE). These cocrystals undergo up to a quantitative [2 + 2] cycloaddition reaction in the organic solid state upon exposure to ultraviolet light. Utilizing the ability of these chlorinated anilines to engage in both N–H···N hydrogen bonds along with homogeneous and face-to-face π–π stacking interactions ultimately positions BPE in a suitable location to photoreact and generate the stereoselective photoproduct *rctt*-tetrakis­(4-pyridyl)­cyclobutane (TPCB). The tendencies for these chlorinated anilines to form homogeneous π-stacks were investigated by means of density functional theory calculations with the goal to determine not only the overall strength but also the preference for this stacking pattern. In addition, a series of isostructural cocrystals were also achieved by incorporating two isosteric hydrogen-bond acceptors, namely 1,2-bis­(4-pyridyl)­acetylene (BPA) and azobipyridine (Azo), with these chlorinated anilines.

## Graphical abstract

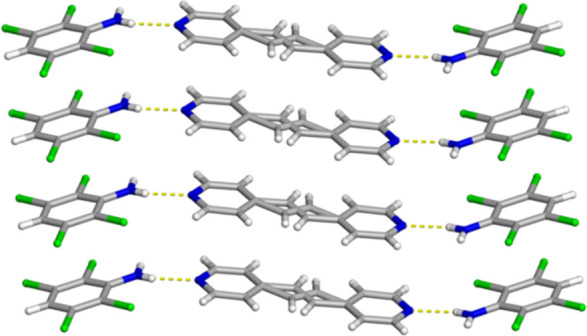

## Introduction

The design of molecular cocrystals that have predictable chemical and physical properties remains a central goal for crystal engineers and materials scientists.^1,2^ In general, these multicomponent solids are realized by exploiting noncovalent interactions found between complementary donor and acceptor sites on the constituent molecules that favor cocrystal formation.^3,4^ An understanding of these noncovalent interactions, along with their hierarchy, is critical to control the overall crystal structure along with their properties. In particular, hydrogen and halogen bonding continues to be highly utilized and reliable noncovalent interactions in the formation of these cocrystals.^5,6^

The field of solid-state photochemistry, especially the light-induced [2 + 2] cycloaddition reaction, has embraced cocrystal formation as a means to overcome issues of crystal packing; since, most reactants are photostable as a single-component solid.^7^ The introduction of the second component (i.e., cocrystal former) utilizes noncovalent interactions to position the carbon–carbon double bond (C=C) on a reactant molecule in a suitable location to photoreact.^8–10^ In particular, chemists have utilized hydrogen^11,12^ and halogen^13,14^ bonding interactions to aid in the formation of these photoreactive multicomponent solids. The advantage of performing these cycloaddition reactions in solids, over a solution-based approach, is the ability to achieve higher yields and stereospecific products due to the constrained environment within the crystal.^15^

Initially, this research group investigated 1,4-diiodoperchlorobenzene as a molecular template, since we could take advantage of its ability to engage in both I···N halogen bonds and homogeneous and face-to-face π–π stacking interactions.^16^ The combination of these noncovalent forces positioned a pair of reactant molecules in a suitable orientation and distance to undergo a [2 + 2] cycloaddition reaction.^17^ Still today, a focus for this group has been the utilization of chlorinated benzenes as molecular templates to achieve photoreactions in molecular solids.^18–20^ In particular, we have also reported that iodoperchlorobenzene,^21^ 1,2,4,5-tetrachloro-3-iodobenzene,^22^ and 2,4,6-trichlorophenol^23^ will template photoreactions by taking advantage of their halogen and/or hydrogen bonding capabilities along with their tendencies to engage in homogeneous and face-to-face π–π stacking interactions.^24^

With the goal to expand this research, we anticipated the addition of an amine group to a chlorinated benzene would result in a suitable template to achieve a [2 + 2] cycloaddition reaction. Using this as inspiration, herein, we report the ability to form a pair of photoreactive cocrystals containing either 2,3,5,6-tetrachloroaniline (C_6_H_3_Cl_4_N) or 2,4,6-trichloroaniline (C_6_H_4_Cl_3_N) with *trans*-1,2-bis­(4-pyridyl)­ethylene (BPE) (Scheme 1). To the best of our knowledge, this contribution is the first reported example of utilizing chlorinated anilines as molecular templates to achieve a series of photoreactions within organic cocrystals. These components self-assemble to form a pair of three-component hydrogen-bonded solids, namely 2­(C_6_H_3_Cl_4_N)·(BPE) and 2­(C_6_H_4_Cl_3_N)·(BPE), that are held together by the combination of N–H···N hydrogen bonds and homogeneous π–π stacking forces. These noncovalent interactions position a pair of BPE molecules in an appropriate position to photoreact.^17^ Upon exposure to ultraviolet light, the cocrystals undergo up to a quantitative yield for the stereoselective cycloaddition reaction to produce the stereospecific product *rctt*-tetrakis­(4-pyridyl)­cyclobutane (TPCB) (Scheme 2).

**Scheme 1:**
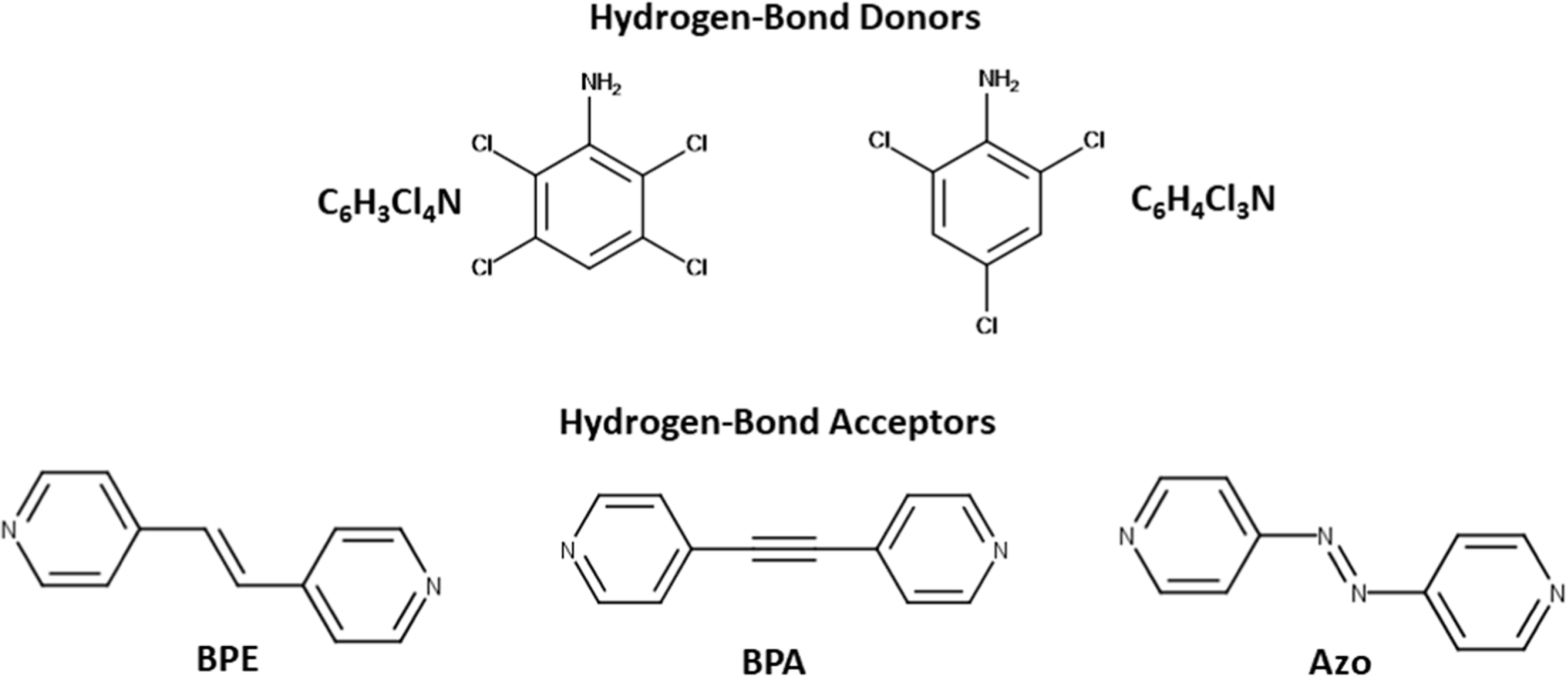
Structures of the Hydrogen-Bond Donors and Acceptors within the Various Cocrystals

**Scheme 2:**
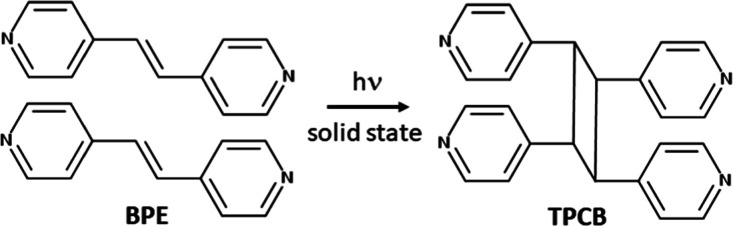
Structure of the Solid-State [2 + 2] Cycloaddition Reaction of BPE to Produce TPCB

To explore the reliability of these anilines to form additional isostructural cocrystals, two different isosteric hydrogen-bond bipyridine-based acceptors were also investigated, namely 1,2-bis­(4-pyridyl)­acetylene (BPA) and 4,4′-azopyridine (Azo) (Scheme 1). Lastly, the strength and selectivity of the face-to-face and homogeneous stacking pattern was examined by means of a series of density functional theory (DFT) calculations. These calculations determined the binding energies for the observed homogeneous and theoretical heterogeneous patterns that illustrate the preference in the stacking arrangement for these anilines in the solid state.

## Results and Discussion

### Structure and Photoreactivity of 2,3,5,6-Tetrachloroaniline Cocrystals

The components of 2­(C_6_H_3_Cl_4_N)·(BPE) crystallizes in the centrosymmetric monoclinic space group *P*2_1_/*c*. The asymmetric unit contains a whole molecule of C_6_H_3_Cl_4_N along with half a molecule of BPE where inversion symmetry generates the remainder of the molecule. The cocrystal is sustained by N–H···N hydrogen bonds [N···N 2.978(2) Å] that results in a discrete three-component hydrogen-bonded assembly (Figure 1). Curiously, the second N–H bond does not interact with any suitable hydrogen-bond acceptor within 2­(C_6_H_3_Cl_4_N)·(BPE). The ethylene bridge within BPE is found to be disordered over two positions at 290 K. A free-variable refinement determined the occupancy factors for the disordered olefin refined to values of 0.83:0.17. To determine the nature of the crystallographic disorder, a second complete data set was collected on the same crystal at 100 K. Again, the occupancy factors for the disorder were calculated by means of a free-variable refinement, which now returned a value of 0.92:0.08. The change in occupancy factors at different temperatures confirms that the disorder is dynamic in nature and the ethylene group undergoes a pedal-like motion in the solid state.^25^ This type of dynamic disorder has been widely reported in similarly shaped bipyridine-based molecules.^26^ The hydrogen-bond donor and acceptor are found to lie relatively close to coplanarity, with a value of 16.86° at 290 K (Figure 1). Neighboring three-component hydrogen-bonded assemblies interact via C–H···Cl interactions [C···Cl 3.847(2) Å] that generate a one-dimensional wave-like structure (Figure 2).^27^

**Figure 1:**
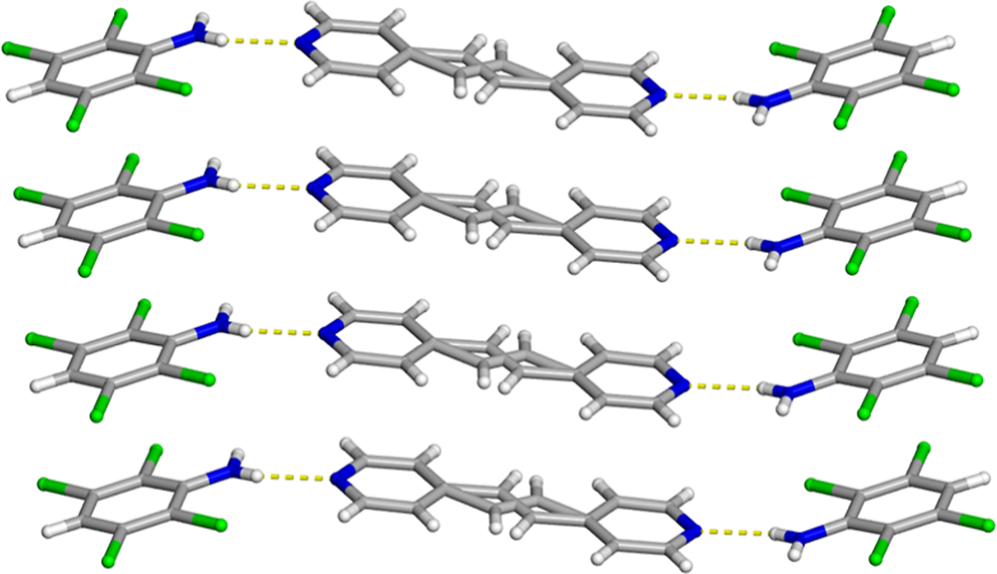
X-ray crystal structure of 2­(C_6_H_3_Cl_4_N)·(BPE) illustrating the infinite face-to-face and homogeneous π–π stacking pattern of the aromatic rings. The N–H···N hydrogen bonds are shown as yellow dashed lines.

**Figure 2:**
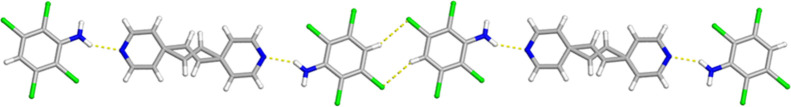
X-ray crystal structure of 2­(C_6_H_3_Cl_4_N)·(BPE) illustrating the one-dimensional wave-like structure. The N–H···N hydrogen bonds and C–H···Cl contacts are shown as yellow dashed lines.

Important to the photochemical reactivity of this cocrystal, both aromatic molecules are found to engage in homogeneous and face-to-face π–π stacking interactions. These infinite stacks have a centroid-to-centroid distance of 3.8033(2) Å equal to the crystallographic *b*-axis and well within the accepted limit for a photoreaction.^17^ Due to translational symmetry, the disordered ethylene group, within the infinite stack, is parallel and with the confirmed pedal motion of BPE, enhances its ability to undergo a solid-state [2 + 2] cycloaddition reaction.^28^

To determine the photoreactivity of 2­(C_6_H_3_Cl_4_N)·(BPE), a dried powdered sample was exposed to ultraviolet radiation in a photochemical cabinet from a 450 W medium-pressure mercury vapor bulb. A [2 + 2] cycloaddition was observed by using ^1^H nuclear magnetic resonance spectroscopy (^1^H NMR) by the nearly complete loss of the olefinic peak at 7.55 ppm on BPE with the concomitant appearance of a peak at 4.67 ppm that corresponds to the hydrogens on the cyclobutane ring within TPCB (Figures S1 and S2).^29–31^ The yield for the solid-state [2 + 2] cycloaddition reaction was determined to be 96% after 70 h of exposure.

With the goal of determining the structure of the bulk solid and comparing it to the single-crystal structure of 2(C_6_H_3_Cl_4_N)·(BPE), powder X-ray diffraction (PXRD) experiment was performed. The resulting diffractogram confirms that the resulting solid material matches the reported cocrystal structure based on its calculated powder pattern (Figure S5). This level of purity for the bulk solid, when compared with the single-crystal data, supports the observed near quantitative yield for the photoreaction.

A pair of isostructural cocrystals, namely 2­(C_6_H_3_Cl_4_N)·(BPA) and 2­(C_6_H_3_Cl_4_N)·(Azo), were realized with similar crystallographic parameters due to the inclusion of the two different isosteric bipyridines (Table 1). As seen before, the cocrystals are held together primarily by N–H···N hydrogen bonds [N···N (Å): BPA 2.994(7) and Azo 3.013(3)] to yield isostructural three-component assemblies (Figure 3). Again, the second N–H group is not hydrogen bonding with any suitable acceptor within either cocrystal. The bridging azo group within 2­(C_6_H_3_Cl_4_N)·(Azo) is found to be disordered and after a free-variable refinement returned a value of 0.81:0.19 at 290 K (Figure 3b). Similar to 2­(C_6_H_3_Cl_4_N)·(BPE), the aromatic donor and acceptor are close to coplanarity with a value of 14.55° for 2­(C_6_H_3_Cl_4_N)·(BPA) and 16.77° for 2­(C_6_H_3_Cl_4_N)·(Azo) (Figure 3). Again, these hydrogen-bonded assemblies interact with a neighbor by C–H···Cl contacts [C···Cl (Å): BPA 3.812(6) and Azo 3.818(2)] that generate a one-dimensional wave-like structure (Figure 4).^27^ Again, both aromatic rings are π–π stacking in a face-to-face and homogeneous arrangement that runs along the crystallographic *b*-axis with distances of 3.8329(4) and 3.8365(4) Å for 2­(C_6_H_3_Cl_4_N)·(BPA) and 2­(C_6_H_3_Cl_4_N)·(Azo), respectively (Table 1).

**Table 1:** Unit Cell Data for the Three Isostructural Cocrystals Based upon C_6_H_3_Cl_4_N at 290 K

Cocrystal	2(C_6_H_3_Cl_4_N)·(BPE)	2(C_6_H_3_Cl_4_N)·(BPA)	2(C_6_H_3_Cl_4_N)·(Azo)
crystal system	monoclinic	monoclinic	monoclinic
space group	*P*2_1_/*c*	*P*2_1_/*c*	*P*2_1_/*c*
*a* (Å)	13.5562(11)	13.4496(10)	13.5495(14)
*b* (Å)	3.8759(3)	3.8329(4)	3.8365(4)
*c* (Å)	25.3818(18)	25.455(2)	25.335(2)
α (°)	90	90	90
β (°)	99.503(3)	98.123(3)	100.627(4)
γ (°)	90	90	90
*V* (Å^3^)	1315.32(17)	1299.1(2)	1294.4(2)

**Figure 3:**
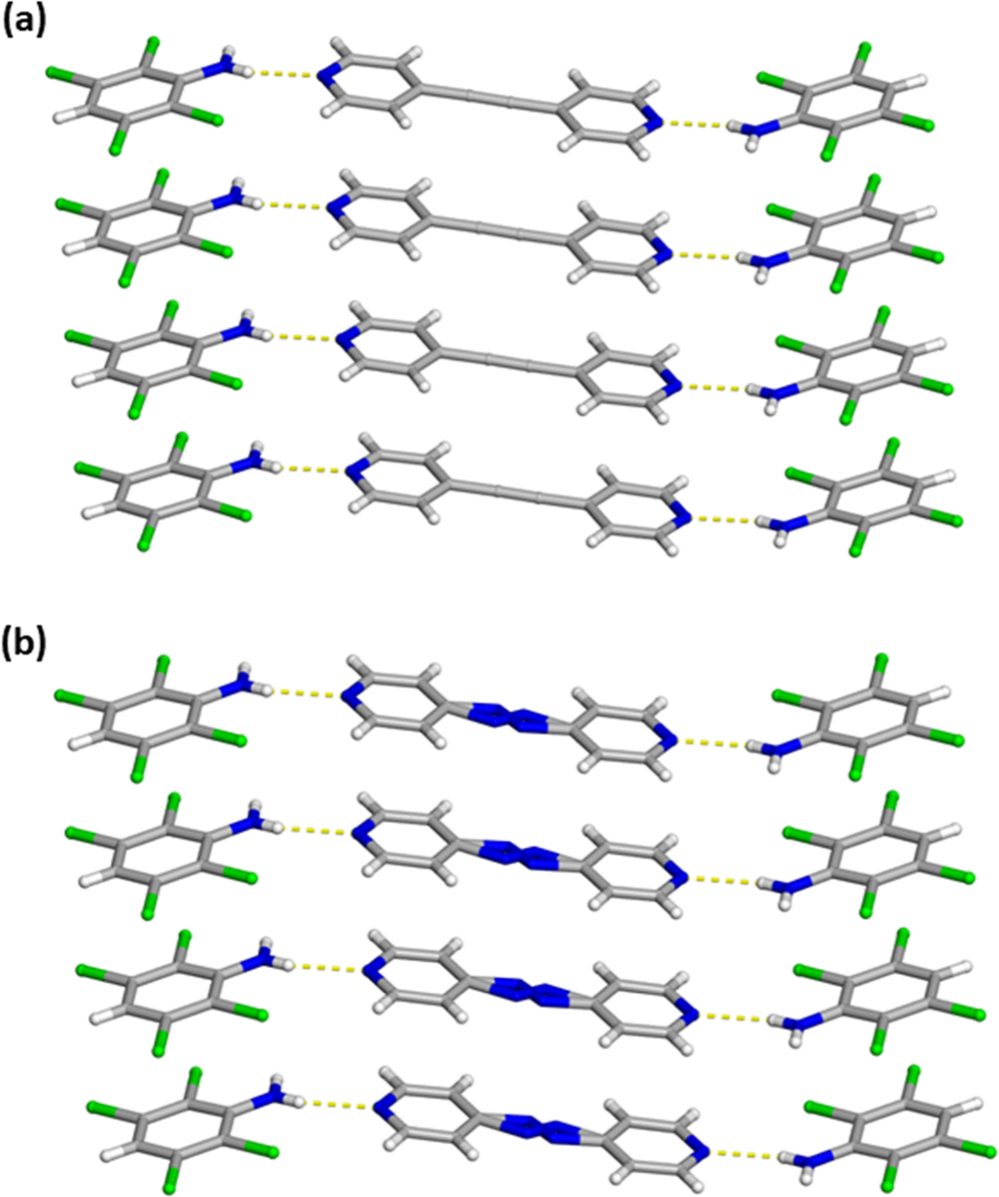
X-ray crystal structure of (a) 2­(C_6_H_3_Cl_4_N)·(BPA) and (b) 2­(C_6_H_3_Cl_4_N)·(Azo) illustrating the isostructural features along with the infinite face-to-face and homogeneous π–π stacking pattern of the aromatic rings. The N–H···N hydrogen bonds are shown as yellow dashed lines.

**Figure 4:**
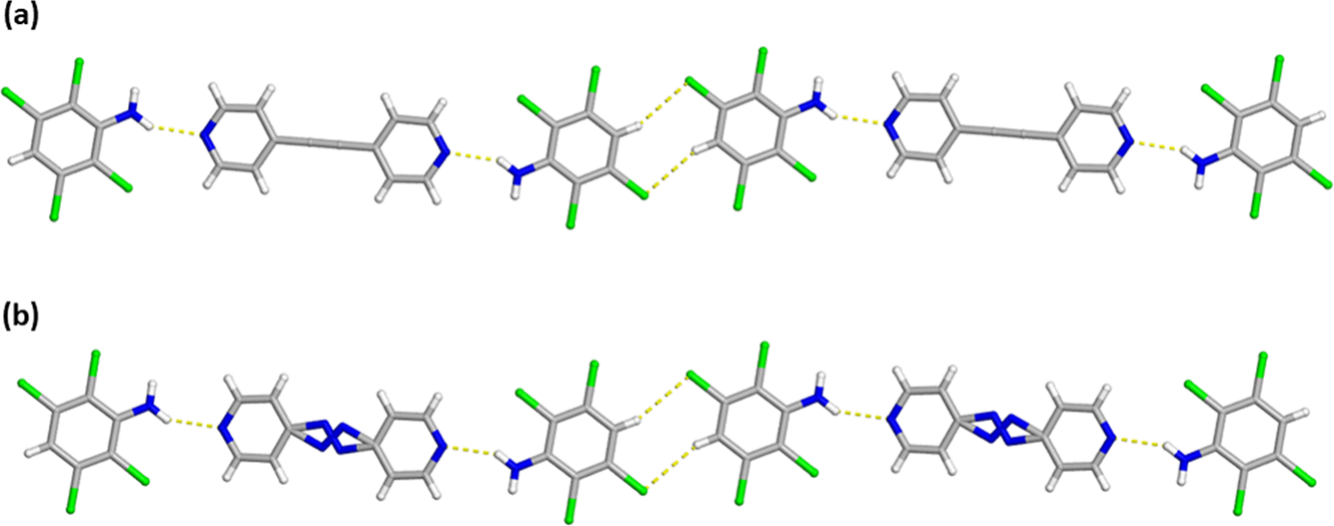
X-ray crystal structure of (a) 2­(C_6_H_3_Cl_4_N)·(BPA) and (b) 2­(C_6_H_3_Cl_4_N)·(Azo) illustrating the isostructural features of the one-dimensional wave-like structures. The N–H···N hydrogen bonds and C–H···Cl contacts are shown as yellow dashed lines.

To discern the bulk structure and purity of these two solids, a pair of PXRD experiments were performed. Each of the resulting diffractograms were in good agreement with the calculated powder pattern for both 2­(C_6_H_3_Cl_4_N)·(BPA) and 2­(C_6_H_3_Cl_4_N)·(Azo) (Figures S6 and S7). Again, a high level of purity was achieved for these isostructural cocrystals when comparing the observed peaks to the theoretical powder pattern based upon the single-crystal diffraction data.

### Structure and Photoreactivity of 2,4,6-Trichloroaniline Cocrystals

The molecular components of the cocrystal 2­(C_6_H_4_Cl_3_N)·(BPE) crystallize in the centrosymmetric triclinic space group *P*1̅. Similar to the case before, a whole molecule of C_6_H_4_Cl_3_N along with half a molecule of BPE is found within the asymmetric unit. The application of an inversion operation produces the remainder of the molecule and a second C_6_H_4_Cl_3_N. Again, the three-component assembly is held together by N–H···N hydrogen bonds [N···N 3.177(2) Å] (Figure 5). Similar to the previous cocrystals, the second N–H bond in 2­(C_6_H_4_Cl_3_N)·(BPE) is not hydrogen bonding with any suitable acceptor group within the solid. Surprisingly, the ethylene group within BPE is found to be ordered at 290 K. The aromatic rings within the hydrogen-bonded assembly are found to be twisted at a much larger angle than that in 2­(C_6_H_3_Cl_4_N)·(BPE) with a value of 43.16° (Figure 5). Due to this twisting, neighboring hydrogen-bonded assemblies, within the infinite stack, interact via C–H···Cl [C···Cl 3.704(2) Å] interactions involving an *ortho*-chlorine to the amine and an α-hydrogen on the pyridine ring (Figure 5).^27^ A reasonable explanation for this ordered ethylene group within 2(C_6_H_3_Cl_4_N)·(BPE) could be attributed to these C–H···Cl interactions that hinder the pyridine ring to move in the crystal lattice, which is required to achieve pedal motion.

**Figure 5:**
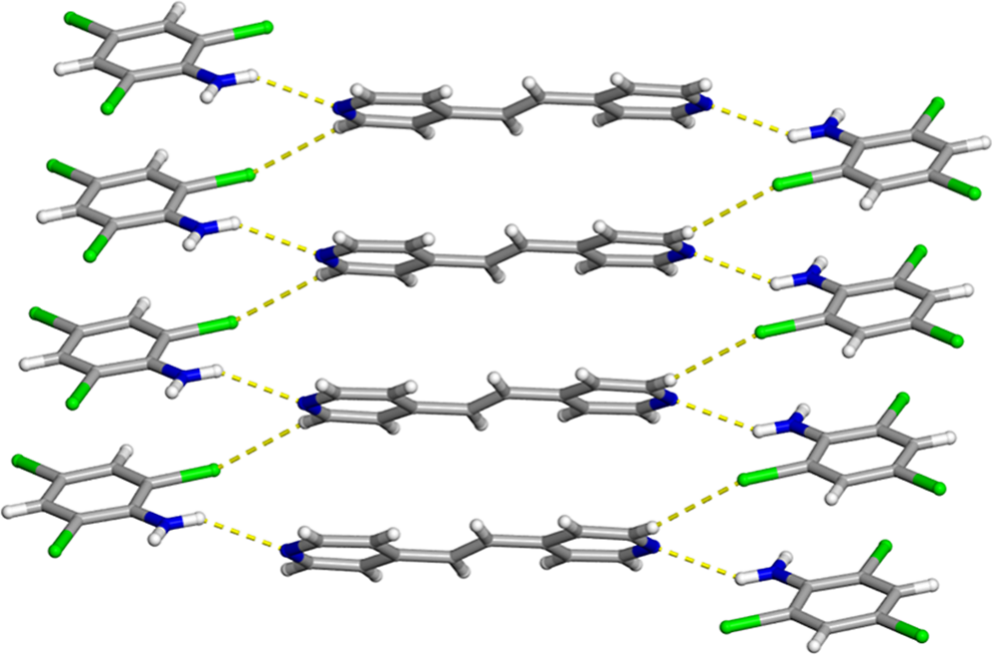
X-ray crystal structure of 2­(C_6_H_4_Cl_3_N)·(BPE) illustrating the infinite face-to-face and homogeneous π–π stacking pattern of the aromatic rings. The N–H···N hydrogen bonds and C–H···Cl contacts are shown as yellow dashed lines.

As expected, both the hydrogen-bond donor and acceptor are found to engage in homogeneous and face-to-face π–π stacking interactions within 2­(C_6_H_4_Cl_3_N)·(BPE). These infinite stacks run along the crystallographic *a*-axis with a distance of 3.8661(6) Å. Due to translational symmetry, the photoreactive C=C centers are found parallel and within an appropriate distance for a [2 + 2] cycloaddition reaction in the solid state.^17^

The photoreactivity of 2­(C_6_H_4_Cl_3_N)·(BPE) was investigated by taking a dried powder sample and exposing it to ultraviolet light. A [2 + 2] cycloaddition reaction was detected by the complete loss of the olefinic peak on BPE at 7.55 ppm and the appearance of a cyclobutane peak at 4.67 ppm, confirming the formation of the stereoselective photoproduct TPCB by using ^1^H NMR (Figures S3 and S4).^29–31^ Within 70 h of exposure, the solid-state photoreaction reached a quantitative yield. Importantly, the outcome of this cycloaddition reaction is significantly greater than the 89% conversion that MacGillivray and co-workers reported for the cocrystal of BPE with 2,4,6-trichlorophenol, an isosteric donor.^32^

The bulk crystalline material containing 2­(C_6_H_4_Cl_3_N)·(BPE) was also analyzed using PXRD. After data collection, the diffractogram confirmed that the bulk solid matched the calculated powder pattern of the cocrystal (Figure S8). The quantitative yield for the [2 + 2] cycloaddition reaction is in agreement with the purity of the bulk solid, which positions all of the reactant molecules in a suitable location to photoreact.

Again, to test the reliability of C_6_H_4_Cl_3_N to form isostructural cocrystals with BPA and Azo, additional cocrystal experiments were performed. As a result, these isosteric hydrogen-bond acceptors formed two isostructural cocrystals with the formulas 2­(C_6_H_4_Cl_3_N)·(BPA) and 2­(C_6_H_4_Cl_3_N)·(Azo) (Table 2). These cocrystals are once again held together by N–H···N hydrogen bonds [N···N (Å): BPA 3.166(3) and Azo 3.202(2)] to yield similar three-component assemblies (Figure 6). Similar to all of the previous cocrystals, the second N–H group is not interacting with any suitable hydrogen-bond acceptor group within either solid. The azo group within 2­(C_6_H_4_Cl_3_N)·(Azo) is found to be ordered at 290 K (Figure 6b). As expected, the aromatic rings are engaged in face-to-face and homogeneous π–π stacking interactions that lie along the crystallographic *a*-axis with distances of 3.8472(3) Å for 2­(C_6_H_4_Cl_3_N)·(BPA) and 3.8567(17) Å for 2­(C_6_H_4_Cl_3_N)·(Azo) (Table 2). In addition, these stacked aromatic rings also interact with nearest neighbors by weak C–H···Cl forces [C···Cl (Å): BPA 3.684(2) and Azo 3.725(2)] (Figure 6).^27^ As seen in 2­(C_6_H_4_Cl_3_N)·(BPE), these C–H···Cl interactions could again be the reason behind the ordered azo group within 2­(C_6_H_4_Cl_3_N)·(Azo).

**Table 2:** Unit Cell Data for the Three Isostructural Cocrystals Based upon C_6_H_4_Cl_3_N at 290 K

Cocrystal	2(C_6_H_4_Cl_3_N)·(BPE)	2(C_6_H_4_Cl_3_N)·(BPA)	2(C_6_H_4_Cl_3_N)·(Azo)
crystal system	triclinic	triclinic	triclinic
space group	*P*1̅	*P*1̅	*P*1̅
*a* (Å)	3.8661(6)	3.8472(3)	3.8567(17)
*b* (Å)	11.4230(18)	11.7667(6)	11.312(4)
*c* (Å)	14.207(3)	13.9604(9)	14.302(6)
α (°)	88.020(5)	88.187(2)	88.082(12)
β (°)	83.395(6)	84.818(2)	83.888(14)
γ (°)	82.220(6)	80.673(2)	80.640(13)
*V* (Å^3^)	617.40(17)	620.97(7)	612.1(4)

**Figure 6:**
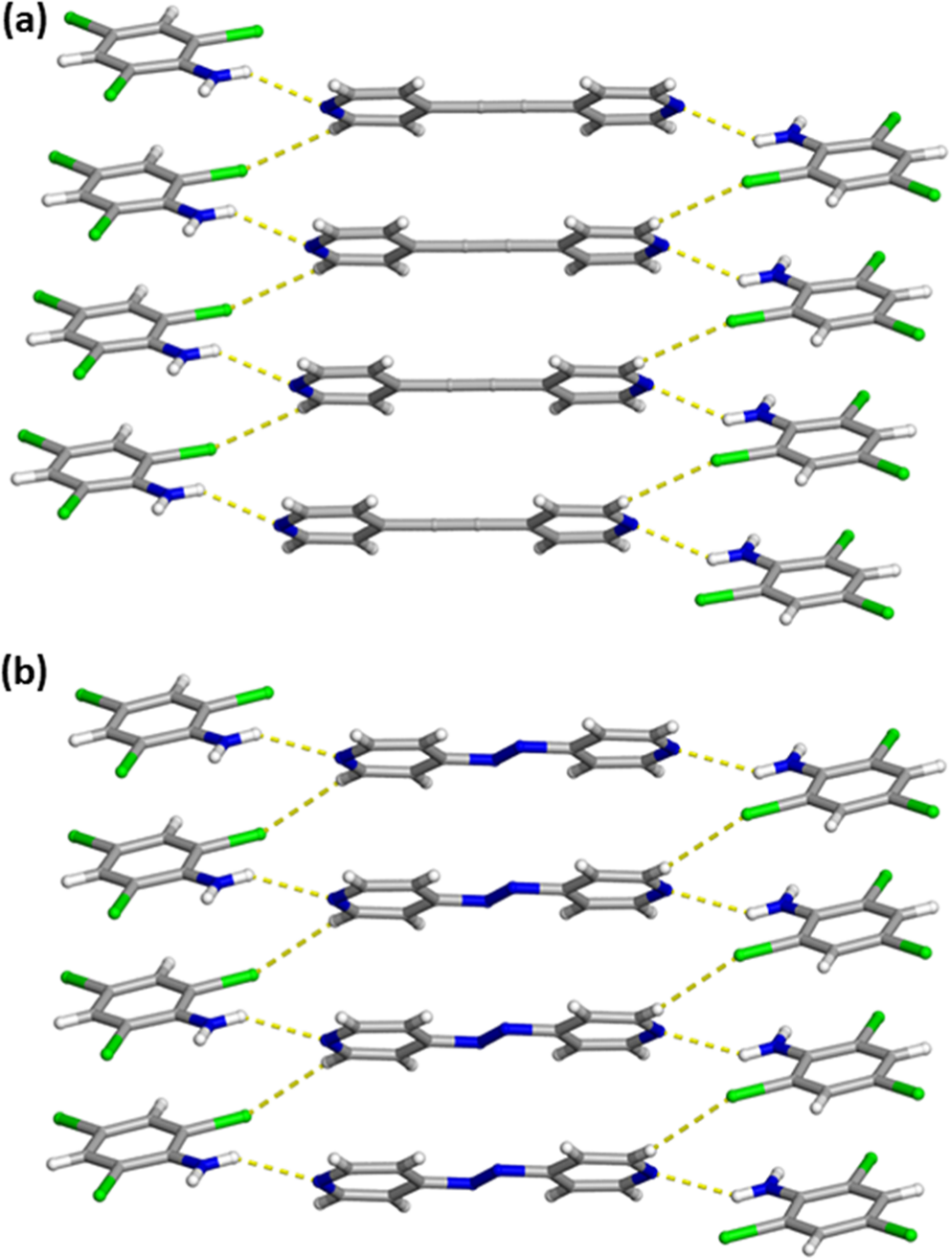
X-ray crystal structure of (a) 2­(C_6_H_4_Cl_3_N)·(BPA) and (b) 2­(C_6_H_4_Cl_3_N)·(Azo) illustrating the isostructural features along with the infinite face-to-face and homogeneous π–π stacking pattern of the aromatic rings. The N–H···N hydrogen bonds and C–H···Cl contacts are shown as yellow dashed lines.

The bulk properties and overall purity of these solids that contained 2­(C_6_H_4_Cl_3_N)·(BPA) and 2­(C_6_H_4_Cl_3_N)·(Azo) were also studied by PXRD experiments. As before, the observed diffractograms are in good agreement with the calculated powder pattern for both cocrystals based upon their single-crystal data (Figures S9 and S10). As a result, the resulting solids once again achieve a high level of purity on the basis of the overlap of the various peaks in the diffractograms.

### Density Functional Theory Calculations for the π–π Stacking Energies

A theoretical study utilizing density functional theory (DFT) at the M062X/aug-cc-pVTZ level of theory was undertaken to quantify the preference of the observed homogeneous over theoretical heterogeneous patterns for both chlorinated aniline donors in a face-to-face orientation. The homogeneous π–π stacking energies for both C_6_H_3_Cl_4_N and C_6_H_4_Cl_3_N were calculated using atomic positions determined from the single-crystal X-ray diffraction data from the corresponding cocrystal. The counterpoise corrected binding energy for the face-to-face and homogeneous π-stack was determined to be −34.9 and −30.0 kJ/mol for 2­(C_6_H_3_Cl_4_N)·(BPE) and 2­(C_6_H_4_Cl_3_N)·(BPE), respectively (Figures S11 and S12). With the goal to determine the preference for the homogeneous over the heterogeneous π-stacking pattern, additional DFT calculations were completed. To determine the energy for this hypothetical stacking arrangement, a mock pyridine ring replaced a chlorinated aniline at identical atomic positions and then the calculation was rerun at the same level of theory. Initially, the nitrogen atom on the pyridine was placed above or syn to the amine group on the aniline (Figures S13 and S14). The heterogeneous corrected π-stack binding energy for the C_6_H_3_Cl_4_N cocrystal was −15.8 kJ/mol and the C_6_H_4_Cl_3_N solid had a value of −20.9 kJ/mol. A second orientation was also investigated by means of DFT calculations, where the nitrogen atom was positioned anti to the amine group. These DFT calculations returned values of −18.2 and −15.1 kJ/mol (Figures S15 and S16) for the C_6_H_3_Cl_4_N and C_6_H_4_Cl_3_N cocrystals, respectively. Comparing these binding energies confirms the preference of the homogeneous π-stack pattern in these cocrystals which is required if these chlorinated anilines are to behave as molecular templates and achieve a photoreaction in the organic solid state.

## Conclusion

In this contribution, we report the ability of both C_6_H_3_Cl_4_N and C_6_H_4_Cl_3_N to behave as a molecular template to achieve a solid-state [2 + 2] cycloaddition reaction involving BPE. The chlorinated anilines align the reactant in a suitable position to photoreact, since it engages in both N–H···N hydrogen bond along with a homogeneous and face-to-face π–π stacking arrangement. Currently, we are investigating these templates with other symmetrical bipyridine-based and unsymmetrical reactants to determine if additional photoreactions would occur.

## Experimental Section

### Materials

The templates 2,3,5,6-tetrachloroaniline (C_6_H_3_Cl_4_N) and 2,4,6-trichloroaniline (C_6_H_4_Cl_3_N) along with the reactant *trans*-1,2-bis­(4-pyridyl)­ethylene (BPE) were all purchased from Sigma-Aldrich Chemical (St. Louis, MO, USA). Reagent grade ethanol was also purchased from Sigma-Aldrich Chemical and was used as received. All crystallization studies were performed in 20 mL scintillation vials.

### General Methods

Photoreactions were conducted using UV radiation from a 450 W medium-pressure mercury lamp in an ACE Glass photochemistry cabinet. Both cocrystals containing BPE (ca 75 mg) were dried and placed between a pair of Pyrex glass plates for irradiation. Single crystals of both 2­(C_6_H_3_Cl_4_N)·(BPE) and 2­(C_6_H_4_Cl_3_N)·(BPE) were placed in the photoreactor to determine whether a single-crystal-to-single-crystal reaction would occur. After the first irradiation cycle, each sample lost crystallinity which is attributed to the formation of the cyclobutane ring and the resulting stress on the crystal due to the photoreaction. The photoreactivity of both cocrystals was determined by using ^1^H NMR spectroscopy. The ^1^H NMR spectrum was collected using a Bruker Ascend Evo 400 MHz spectrometer using DMSO-*d*_6_ as a solvent.

After the photoreaction, both solid samples were dissolved in 3.0 mL of ethanol with the goal to form a cocrystal containing TPCB with a particular aniline. In each case, a collection of suitable crystals were investigated by single-crystal X-ray diffraction which only returned a unit cell that corresponded with the structure of pure TPCB that has been previously reported.^33^ Single-crystal X-ray diffraction data were collected on a Bruker D8 VENTURE DUO diffractometer equipped with an IμS 3.0 microfocus source operated at 75 W (50 kV, 1.5 mA) to generate Mo Kα radiation (λ = 0.71073 Å) with a PHOTON III detector. Powder X-ray diffraction data were collected at room temperature on a Bruker D8 Advance X-ray diffractometer using Cu Kα radiation (λ = 1.54056 Å) between 5° and 40° two-theta.

### Synthesis of Cocrystal Containing 2,3,5,6-Tetrachloroaniline

Cocrystals of 2­(C_6_H_3_Cl_4_N)·(BPE), 2­(C_6_H_3_Cl_4_N)·(BPA), and 2­(C_6_H_3_Cl_4_N)·(Azo) were all achieved by dissolving 63.4 mg of C_6_H_3_Cl_4_N in 1.0 mL of ethanol, which was then combined with a separate 2.0 mL ethanol solution containing either 25.0 mg of BPE, 24.7 mg of BPA, or 25.3 mg of Azo (2:1 mol equiv). Each of the combined solutions was mixed, and then the caps were removed to allow for slow evaporation. Within 2 days and with a significant loss of solvent, single crystals were formed that were suitable for X-ray diffraction experimentation.

### Synthesis of Cocrystal Containing 2,4,6-Trichloroaniline

Cocrystals of 2­(C_6_H_4_Cl_3_N)·(BPE), 2­(C_6_H_4_Cl_3_N)·(BPA), and 2­(C_6_H_4_Cl_3_N)·(Azo) were all realized by dissolving 53.9 mg of C_6_H_4_Cl_3_N in 1.0 mL of ethanol and then it was combined with a separate 2.0 mL ethanol solution containing either 25.0 mg of BPE, 24.7 mg of BPA, or 25.3 mg of Azo (2:1 mol equiv). All of the resulting solutions were allowed to slowly evaporate. After 2 days and loss of most of the solvent, single crystals suitable for X-ray diffraction study were formed.

### Density Functional Theory Calculations

To obtain π–π stacking binding energies, density functional theory calculations were performed using the M06-2X density functional as implemented in the Gaussian 16 program. X-ray diffraction data were used to determine the positions of all non-hydrogen atoms. The hydrogen coordinates were obtained by performing a molecular mechanics optimization with all other atoms frozen at the X-ray diffraction values. An aug-cc-pVTZ basis set, stored internally in the Gaussian program, was used for all atoms. The binding energies were computed using the counterpoise method, as implemented in Gaussian. This procedure computes the energy as the difference between the energy of the pair and the energies of the separated molecules. In the case of the separated fragments, the energies are computed by using the entire set of orbitals for the molecular pair.

### Solution Studies for the Photoreactions

With the goal to compare and illustrate the advantage of performing these [2 + 2] cycloaddition reactions in solids, a solution-based photoreaction was also investigated. In both cases, the mass of the components along with the volume of ethanol was identical to the cocrystallization approach. The only difference was that the resulting solutions were placed in a 10 mL clear vial with the cap securely attached, which would not allow for slow evaporation of the solvent. Then, the two vails were placed in the photoreactor and exposed to ultraviolet light from the mercury vapor bulb for 35 h. After drying the exposed solutions, the resulting solids were then studied via ^1^H NMR spectroscopy. In particular, the solution containing C_6_H_3_Cl_4_N and BPE returned a yield of 1.3% (Figure S17) by comparing the integrals for the olefinic peak on BPE at 7.55 ppm to those of the cyclobutane peak on TPCB at 4.67 ppm. The solution containing C_6_H_4_Cl_3_N and BPE did not have a peak at 4.67 ppm associated with the photoproduct TPCB confirming that no cycloaddition reaction was observed at this particular concentration (Figure S18). These results confirm the clear advantage of performing these photoreactions in a controlled environment within solids rather than the fluid nature of the liquid state.

## Supplementary Material



## Notes

Experimental details, X-ray crystallographic data, ^1^H NMR spectra, powder diffractograms, and DFT calculation details (PDF)

## Notes

The cif files can be obtained free of charge at www.ccdc.cam.ac.uk/conts/retrieving.html or from the Cambridge Crystallo-graphic Data Centre, Cambridge, U.K. with the REF codes 2428190–2428196.

## References

[1] Braga D. (2023). Crystal engineering:
from promise to delivery. Chem. Commun..

[2] Desiraju G. R. (2007). Crystal
Engineering: A Holistic View. Angew. Chem.,
Int. Ed..

[3] Corpinot M. K., Bučar D.-K. (2019). A Practical Guide to the Design of
Molecular Crystals. Cryst. Growth Des..

[4] Mukherjee A. (2015). Building upon
Supramolecular Synthons: Some Aspects of Crystal Engineering. Cryst. Growth Des..

[5] Christopherson J.-C., Topić F., Barrett C. J., Friščić T. (2018). Halogen-Bonded
Cocrystals as Optical Materials: Next-Generation Control over Light–Matter
Interactions. Cryst. Growth Des..

[6] Cavallo G., Metrangolo P., Milani R., Pilati T., Priimagi A., Resnati G., Terraneo G. (2016). The Halogen Bond. Chem. Rev..

[7] MacGillivray L. R. (2008). Organic
Synthesis in the Solid State via Hydrogen-Bond-Driven Self-Assembly. J. Org. Chem..

[8] Kole G. K., Mir M. H. (2022). Isolation of elusive
cyclobutane ligands via a template-assisted
photochemical [2 + 2] cycloaddition reaction and their utility in
engineering crystalline solids. CrystEngComm.

[9] Gan M.-M., Yu J.-G., Wang Y.-Y., Han Y.-F. (2018). Template-Directed
Photochemical [2 + 2] Cycloaddition in Crystalline Materials: A Useful
Tool to Access Cyclobutane Derivatives. Cryst.
Growth Des..

[10] Biradha K., Santra R. (2013). Crystal engineering of topochemical
solid state reactions. Chem. Soc. Rev..

[11] Yelgaonkar S. P., Swenson D. C., MacGillivray L. R. (2020). Supramolecular chemistry under mechanochemical
conditions: a small molecule template generated and integrated into
a molecular-to-supramolecular and back-to-molecular cascade reaction. Chem. Sci..

[12] Li C., Swenson D. C., MacGillivray L. R. (2022). Programming
Rapid Functional Group
Diversification into a Solid-State Reaction: Aryl Nitriles for Self-Assembly,
Click Reactivity, and Discovery of Coexisting Supramolecular Synthons. Chem.—Eur. J..

[13] Quentin J., Reinheimer E. W., MacGillivray L. R. (2022). Halogen-Bond Mediated [2 + 2] Photodimerizations:
À la Carte Access to Unsymmetrical Cyclobutanes in the Solid
State. Molecules.

[14] Quentin J., MacGillivray L. R. (2020). Hydrogen-
and Halogen-Bonded Binary Cocrystals with
Ditopic Components: Systematic Structural and Photoreactivity Properties
That Provide Access to a Completed Series of Symmetrical Cyclobutanes. Cryst. Growth Des..

[15] Li C., MacGillivray L. R. (2025). Supramolecular
Matter Through Crystal Engineering:
Covalent Bond Formation to Postsynthetic Modification. Chem.—Eur. J..

[16] Bosch E., Kruse S. J., Krueger H. R., Groeneman R. H. (2019). Role of
π–π Stacking and Halogen Bonding by 1,4-Diiodoperchlorobenzene
To Organize the Solid State To Achieve a [2 + 2] Cycloaddition Reaction. Cryst. Growth Des..

[17] Schmidt G. M. J. (1971). Photodimerization
in the Solid State. Pure Appl. Chem..

[18] Holdaway J., Bosch E., Unruh D. K., Groeneman R. H. (2024). Supramolecular
Catalysis of a Halogen-Bonded Co-crystal that Undergoes a [2 + 2]
Cycloaddition Reaction Formed via Dry Vortex Grinding. Cryst. Growth Des..

[19] Andren M., Bosch E., Krueger H. R., Groeneman R. H. (2024). Achieving
a series of solid-state [2 + 2] cycloaddition reactions involving
1,2-bis­(2-pyridyl)­ethylene within halogen-bonded co-crystals. CrystEngComm.

[20] Powell C. J., Bosch E., Krueger H. R., Groeneman R. H. (2023). Preference
of halogen bonds over hydrogen bonds within a discrete three-component
co-crystal that undergo a [2 + 2] cycloaddition reaction. New J. Chem..

[21] Shapiro N. M., Bosch E., Unruh D. K., Krueger H. R., Groeneman R. H. (2021). Iodoperchlorobenzene
acts as a dual halogen-bond donor to template a [2 + 2] cycloaddition
reaction within an organic co-crystal. CrystEngComm.

[22] Bosch E., Powell C. J., Krueger H. R., Groeneman R. H. (2023). Photoreactive
polymorphic cocrystals utilizing a molecular template with dual halogen
and hydrogen bonding capabilities. Cryst. Growth
Des..

[23] Andren M., Unruh D. K., Krueger H. R., Groeneman R. H. (2024). Expanding
the versatility of trihalophenols as molecular templates to achieve
a series of [2 + 2] cycloaddition reaction involving 1,2-bis­(2-pyridyl)­ethylene. New J. Chem..

[24] Reddy C. M., Kirchner M. T., Gundakaram R. C., Padmanabhan K. A., Desiraju G. R. (2006). Isostructurality, Polymorphism and
Mechanical Properties
of Some Hexahalogenated Benzenes: The Nature of Halogen···Halogen
Interactions. Chem.—Eur. J..

[25] Harada J., Ogawa K. (2009). Pedal motion in crystals. Chem. Soc. Rev..

[26] Harada J., Ogawa K. (2014). What Molecules Are
Likely or Unlikely To Undergo Pedal Motions in
Crystals?. Cryst. Growth Des..

[27] Hathwar V. R., Roopan S. M., Subashini R., Khan F. N., Guru
Row T. N. (2010). Analysis of Cl···Cl and C-H···Cl intermolecular
interactions involving chlorine in substituted 2-chloroquinoline derivatives. J. Chem. Sci..

[28] Elacqua E., Kaushik P., Groeneman R. H., Sumrak J. C., Bučar D.-K., MacGillivray L. R. (2012). A Supramolecular
Protecting Group Strategy Introduced
to the Organic Solid State: Enhanced Reactivity through Molecular
Pedal Motion. Angew. Chem., Int. Ed..

[29] MacGillivray L. R., Reid J. L., Ripmeester J. A. (2000). Supramolecular Control of Reactivity
in the Solid State Using Linear Molecular Templates. J. Am. Chem. Soc..

[30] Papaefstathiou G. S., MacGillivray L. R. (2002). An Inverted
Metal-Organic Framework with Compartmentalized
Cavities Constructed by Using an Organic Bridging Unit Derived from
the Solid State. Angew. Chem., Int. Ed..

[31] Oburn S. M., Elacqua E., Santana C. L., Groeneman R. H. (2020). A diamondoid
net sustained by halogen bonds: employing a cyclobutane to generate
a tetrahedral architecture. CrystEngComm.

[32] Campillo-Alvarado G., Li C., Swenson D. C., MacGillivray L. R. (2019). Application of Long-Range Synthon
Aufbau Modules Based on Trihalophenols to Direct Reactivity in Binary
Cocrystals: Orthogonal Hydrogen Bonding and π–π
Contact Driven Self-Assembly with Single-Crystal Reactivity. Cryst. Growth Des..

[33] Singh A. S., Sun S.-S. (2013). Recyclable nitrate-templated
photochemical [2 + 2]
cycloaddition reaction promoted by a tripodal receptor. Chem. Commun..

