# Supplementary material for: Chlorinated Anilines as Molecular Templates to Achieve [2 + 2] Cycloaddition Reactions within Organic Cocrystals
Source: ACS Omega. 2025 May 21;10(21):21922–8. doi: 10.1021/acsomega.5c01991 (PMC12138658; doi:10.1021/acsomega.5c01991)
Supplement: Supplementary file 1 [file ao5c01991_si_001.pdf]

Supporting Information

for

Chlorinated-anilines as Molecular Templates to achieve [2 + 2] Cycloaddition Reactions within Organic Co-crystals

Grace K. White, Daniel K. Unruh, Herman R. Krueger, Jr. and Ryan H. Groeneman*

Department of Natural Sciences and Mathematics, Webster University, St. Louis, Missouri, USA

Office of the Vice President for Research, University of Iowa, Iowa City, Iowa, USA

1. Single X-ray Diffraction Information and Data Table S2-S5

2. Solid-State ^{1}H NMR Spectroscopic Data S6-S7

3. Powder X-ray Diffraction Data S8-S13

4. Density Functional Theory Calculation Figures S14-S16

5. Solution ^{1}H NMR Spectroscopic Data S17-S18

1. Single X-ray Diffraction Information and Data Table

Data were collected on a Bruker D8 VENTURE DUO diffractometer equipped with a IμS 3.0 microfocus source operated at 75 W (50 kV, 1.5 mA) to generate Mo Kα radiation (λ = 0.71073 Å) and a PHOTON III detector. Crystals were transferred from the vial and placed on a glass slide in Paratone-N oil. A Zeiss Stemi 305 microscope was used to identify a suitable specimen for X-ray diffraction from a representative sample of the material. The selected crystal and a small amount of the oil were collected on a MiTeGen 100-micron MicroLoop and transferred to the instrument. The sample was optically centered with the aid of a video camera to ensure that no translations were observed as the crystal was rotated through all positions. A unit cell collection was then carried out. After it was determined that the unit cell was not present in the CCDC database a data collection strategy was calculated by *APEX6*.[1] The crystal was measured for size, morphology, and color.

After data collection, the unit cell was re-determined using a subset of the full data collection. Intensity data were corrected for Lorentz, polarization, and background effects using the *APEX6*.[1] A numerical absorption correction was applied based on a Gaussian integration over a multifaceted crystal and followed by a semi-empirical correction for adsorption applied using *SADABS*.[1] The program *SHELXT*[2] was used for the initial structure solution and *SHELXL*[3] was used for refinement of the structure. Both programs were utilized within the OLEX2 software.[4] Hydrogen atoms bound to carbon atoms were located in the difference Fourier map and were geometrically constrained using the appropriate AFIX commands.

Table S1. Single-crystal X-ray diffraction data for 2($\mathbf{C_6H_3Cl_4N}$)·(**BPE**) at 290 and 100 K.

compound name	2($\mathbf{C_6H_3Cl_4N}$)·(**BPE**)	2($\mathbf{C_6H_3Cl_4N}$)·(**BPE**)
chemical formula	$C_{24}H_{16}Cl_8N_4$	$C_{24}H_{16}Cl_8N_4$
formula mass	644.01	644.01
crystal system	Monoclinic	Monoclinic
space group	$P2_1/c$	$P2_1/c$
a/Å	13.5562(11)	13.4253(5)
b/Å	3.8759(3)	3.8033(2)
c/Å	25.3818(18)	25.1752(9)
α/°	90	90
β/°	99.503(3)	98.944(1)
γ/°	90	90
V/Å^3	1315.32(17)	1269.83(9)
ρ_{calc} /g cm^{-3}	1.626	1.684
T/K	290	100
Z	2	2
radiation type	MoKα (λ = 0.71073)	MoKα (λ = 0.71073)
absorption coefficient, μ/mm^{-1}	0.880	0.912
no. of reflections measured	30535	22739
no. of independent reflections	3281	3144
R_{int}	0.0360	0.0406
R_1 (I > 2σ(I))	0.0291	0.0255
wR(F^2) (I > 2σ(I))	0.0717	0.0557
R_1 (all data)	0.0353	0.0297
wR(F^2) (all data)	0.0765	0.057
Goodness-of-fit	1.065	1.051
CCDC deposition number	2428190	2428191

Table S2. Single-crystal X-ray diffraction data for 2($\mathbf{C_6H_3Cl_4N}$)·(**BPA**) and 2($\mathbf{C_6H_3Cl_4N}$)·(**Azo**).

compound name	2($\mathbf{C_6H_3Cl_4N}$)·(**BPA**)	2($\mathbf{C_6H_3Cl_4N}$)·(**Azo**)
chemical formula	$C_{24}H_{14}Cl_8N_4$	$C_{22}H_{14}Cl_8N_6$
formula mass	641.99	645.99
crystal system	Monoclinic	Monoclinic
space group	$P2_1/c$	$P2_1/c$
a/Å	13.4496(10)	13.5495(14)
b/Å	3.8329(4)	3.8365(4)
c/Å	25.455(2)	25.335(2)
α/°	90	90
β/°	98.123(3)	100.627(4)
γ/°	90	90
V/Å^3	1299.1(2)	1294.4(2)
ρ_{calc} /g cm^{-3}	1.641	1.657
T/K	290	290
Z	2	2
radiation type	MoKα (λ = 0.71073)	MoKα (λ = 0.71073)
absorption coefficient, μ/mm^{-1}	0.891	0.897
no. of reflections measured	25438	29003
no. of independent reflections	2647	3221
R_{int}	0.0583	0.0479
R_1 (I > 2σ(I))	0.0750	0.0421
wR(F^2) (I > 2σ(I))	0.1692	0.0979
R_1 (all data)	0.0841	0.0499
wR(F^2) (all data)	0.1727	0.1029
Goodness-of-fit	1.180	1.069
CCDC deposition number	2428192	242193

Table S3. Single-crystal X-ray diffraction data for 2($\mathbf{C_6H_4Cl_3N}$)·(**BPE**), 2($\mathbf{C_6H_4Cl_3N}$)·(**BPA**), and 2($\mathbf{C_6H_4Cl_3N}$)·(**Azo**).

compound name	2($\mathbf{C_6H_4Cl_3N}$)·(**BPE**)	2($\mathbf{C_6H_4Cl_3N}$)·(**BPA**)	2($\mathbf{C_6H_4Cl_3N}$)·(**Azo**)
chemical formula	$C_{24}H_{18}Cl_6N_4$	$C_{24}H_{16}Cl_6N_4$	$C_{22}H_{16}Cl_6N_6$
formula mass	575.12	573.11	577.11
crystal system	Triclinic	Triclinic	Triclinic
space group	$P\bar{1}$	$P\bar{1}$	$P\bar{1}$
a/Å	3.8661(6)	3.8472(3)	3.8567(17)
b/Å	11.4230(18)	11.7667(6)	11.312(4)
c/Å	14.207(3)	13.9604(9)	14.302(6)
α/°	88.020(5)	88.187(2)	88.082(12)
β/°	83.395(6)	84.818(2)	83.888(14)
γ/°	82.220(6)	80.673(2)	80.640(13)
V/Å^3	617.40(17)	620.97(7)	612.1(4)
ρ_{calc} /g cm^{-3}	1.547	1.533	1.566
T/K	290	290	290
Z	1	1	1
radiation type	MoKα (λ = 0.71073)	MoKα (λ = 0.71073)	MoKα (λ = 0.71073)
absorption coefficient, μ/mm^{-1}	0.718	0.714	0.727
no. of reflections measured	23725	21819	23980
no. of independent reflections	3086	2538	3010
R_{int}	0.0414	0.0855	0.0420
R_1 (I > 2σ(I))	0.0320	0.0431	0.0331
wR(F^2) (I > 2σ(I))	0.0799	0.1081	0.0817
R_1 (all data)	0.0397	0.0572	0.0412
wR(F^2) (all data)	0.0856	0.1196	0.0875
Goodness-of-fit	1.043	1.039	1.022
CCDC deposition number	2428194	2428195	2428196

2. Solid-State ^{1}H NMR Spectroscopic Data

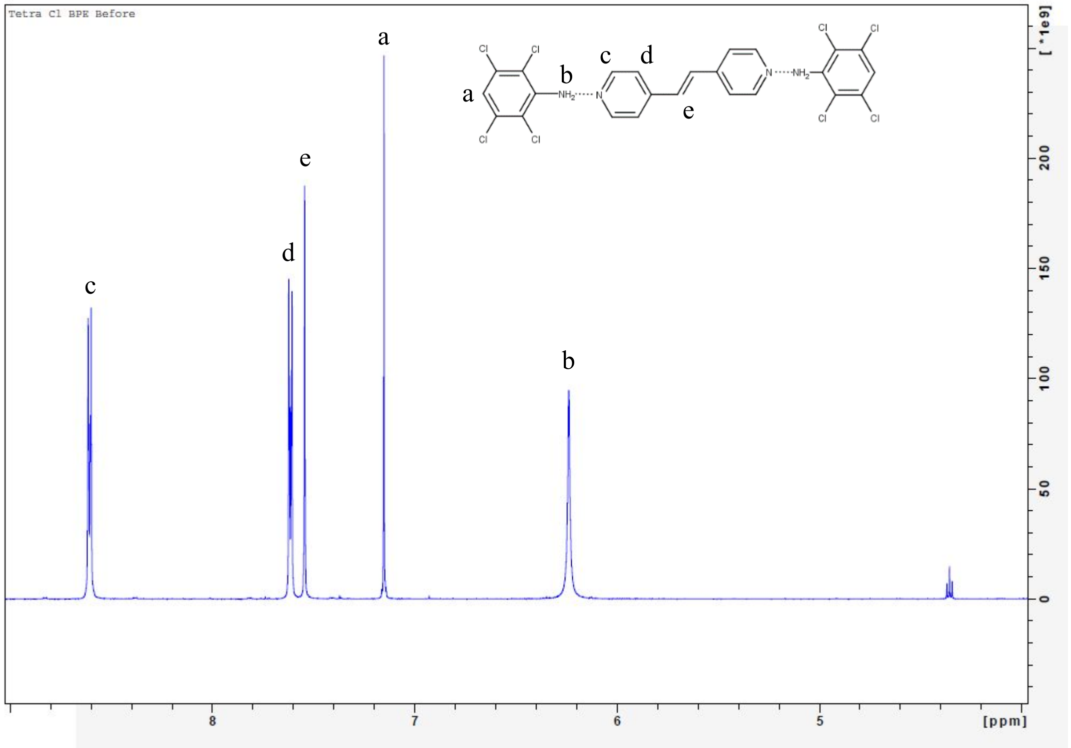

Figure S1: ^{1}H NMR spectrum of the co-crystal 2($\mathbf{C_6H_3Cl_4N}$)·(**BPE**) before UV irradiation (400 MHz, DMSO-d_6).

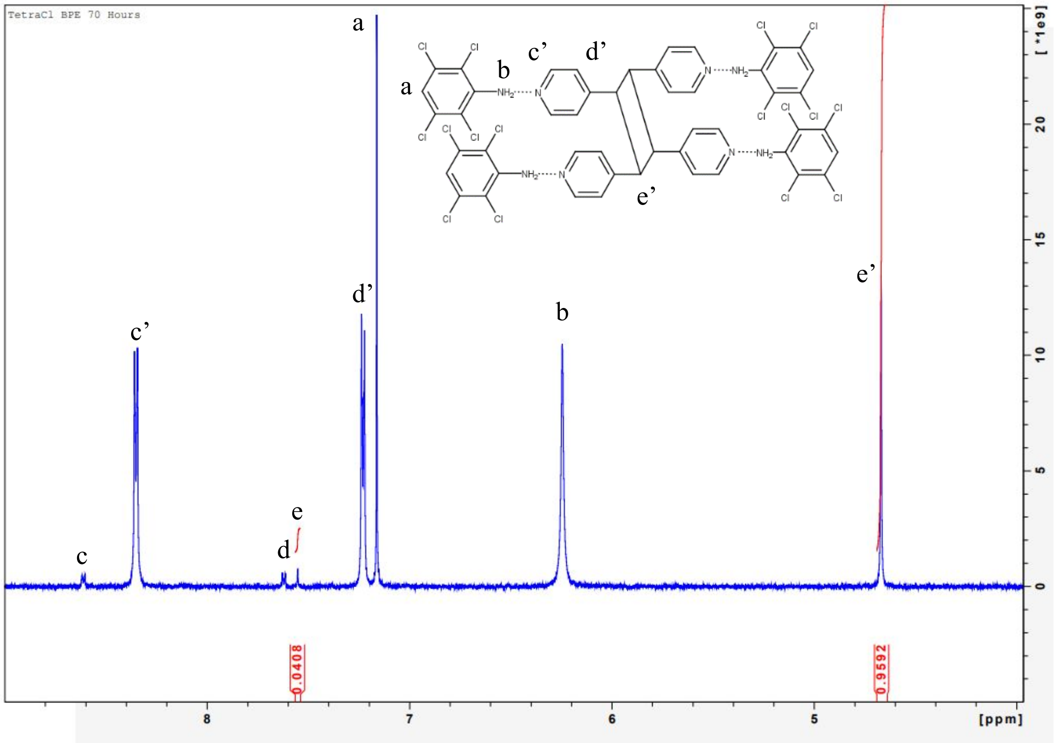

Figure S2: ^{1}H NMR spectrum of the co-crystal 2($\mathbf{C_6H_3Cl_4N}$)·(**BPE**) after 70 hours of UV irradiation reaching a yield of 96% for the [2 + 2] cycloaddition reaction (400 MHz, DMSO-d_6).

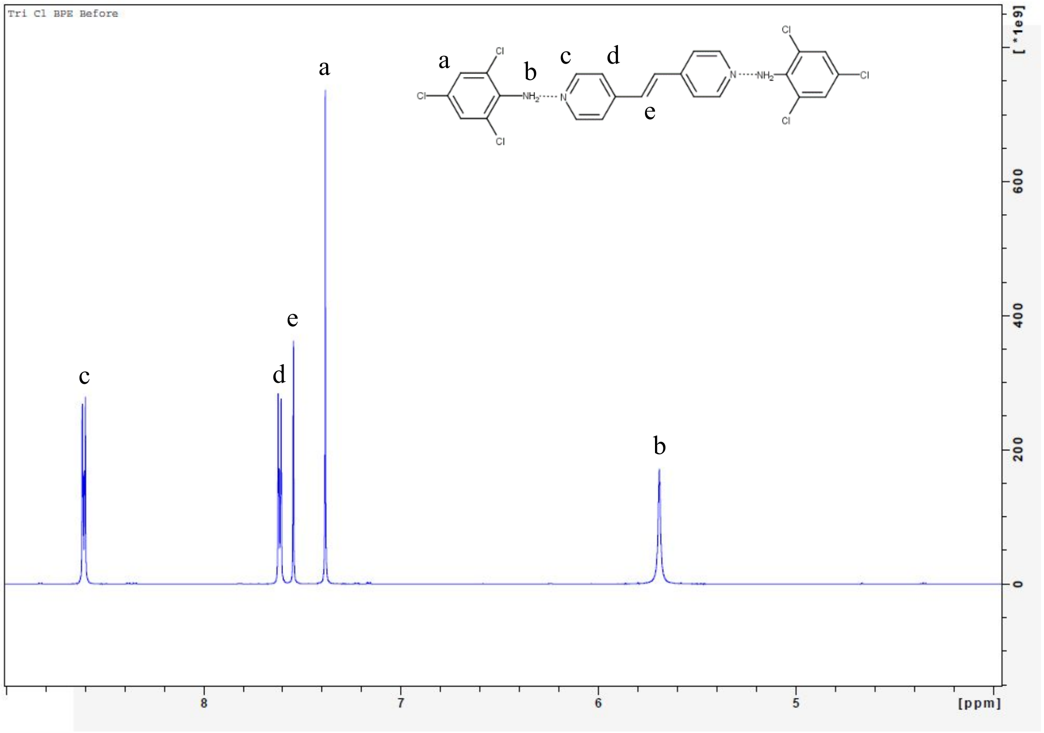

Figure S3: ^{1}H NMR spectrum of the co-crystal 2(**$C_6H_4Cl_3N$**)·(**BPE**) before UV irradiation (400 MHz, DMSO-d_6).

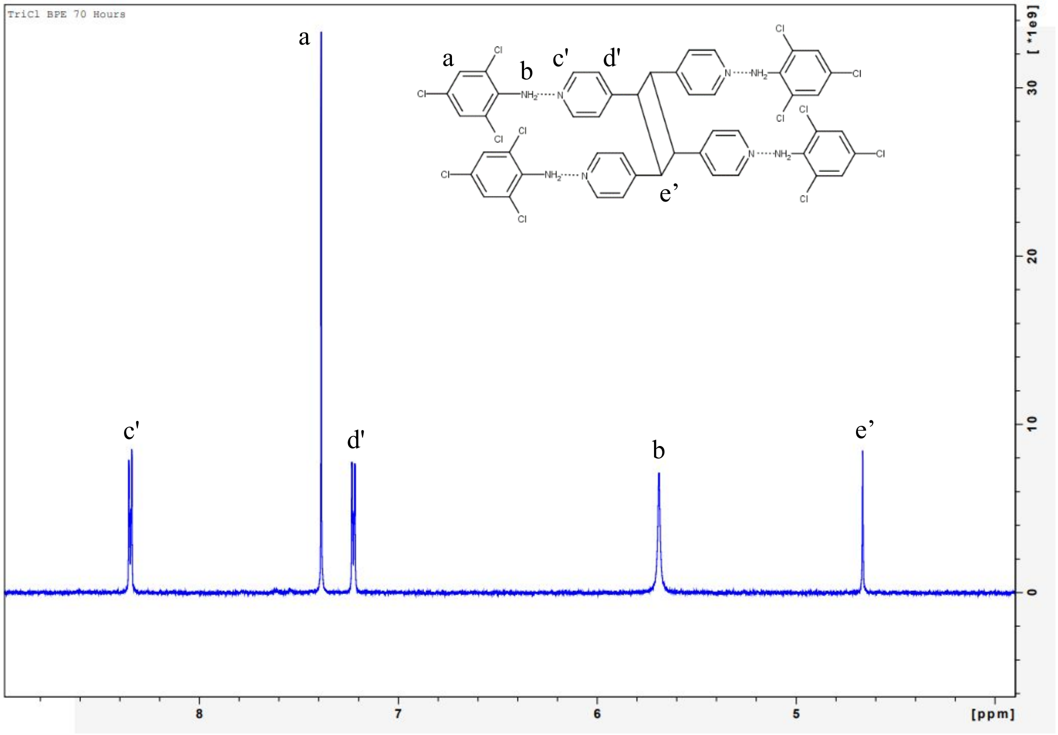

Figure S4: ^{1}H NMR spectrum of the co-crystal 2(**$C_6H_4Cl_3N$**)·(**BPE**) after 70 hours of UV irradiation reaching a quantitative yield for the [2 + 2] cycloaddition reaction (400 MHz, DMSO-d_6).

3. Powder X-ray Diffraction Data

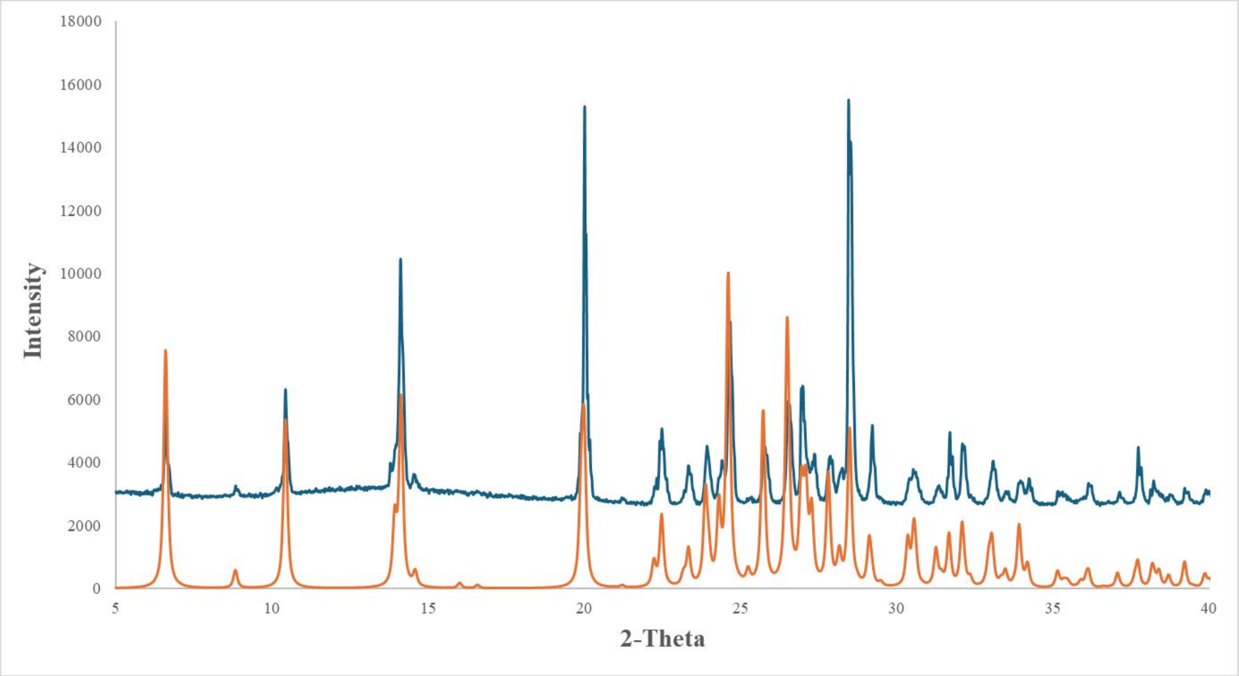

Figure S5. Powder X-ray diffraction data for the bulk sample that contains 2(**$C_6H_3Cl_4N$**)·(**BPE**) (blue) along with the theoretical pattern for 2(**$C_6H_3Cl_4N$**)·(**BPE**) (orange).

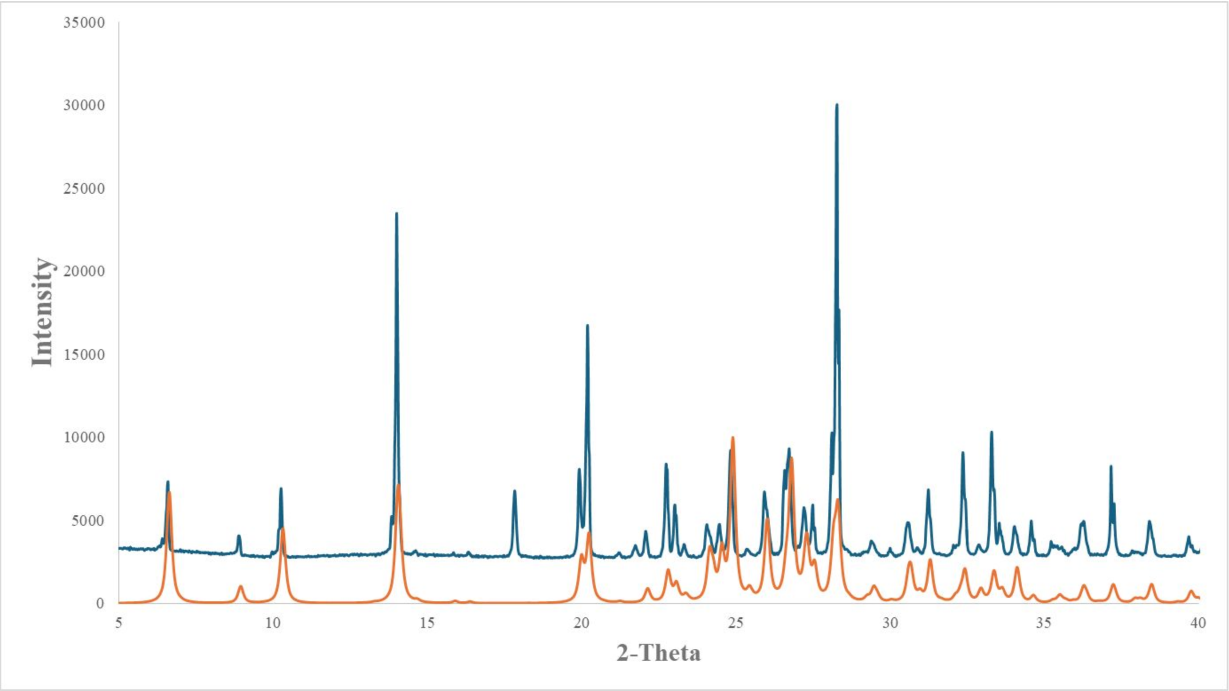

Figure S6. Powder X-ray diffraction data for the bulk sample that contains 2($\mathbf{C_6H_3Cl_4N}$)·(**BPA**) (blue) along with the theoretical pattern for 2($\mathbf{C_6H_3Cl_4N}$)·(**BPA**) (orange).

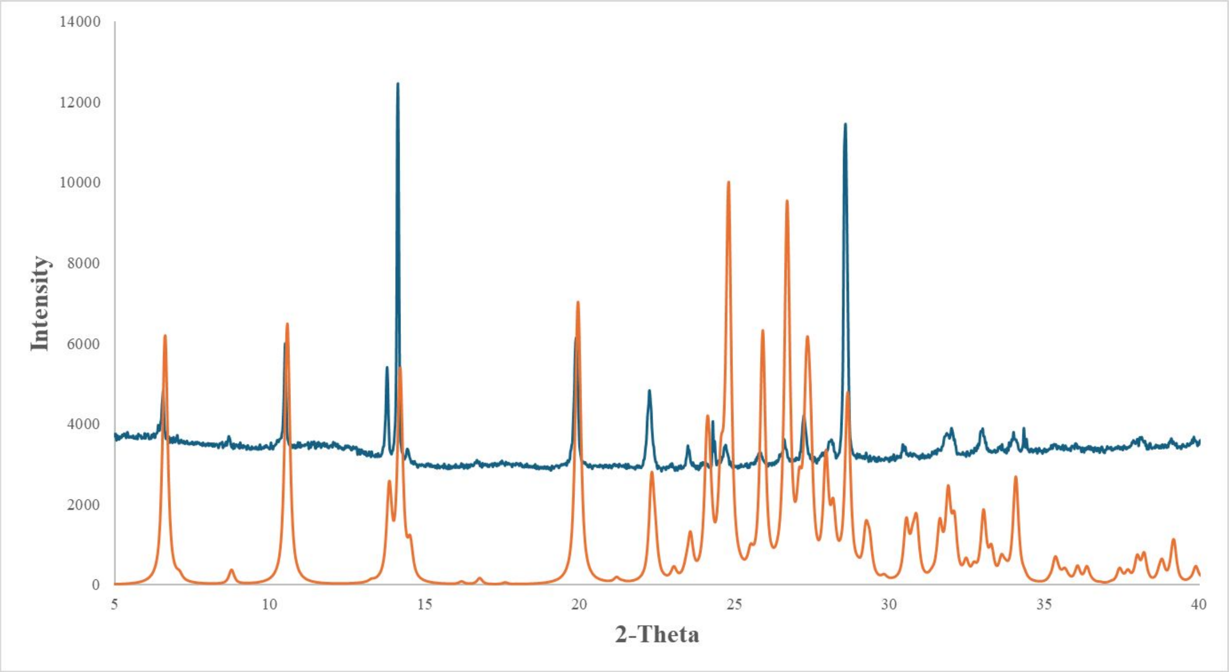

Figure S7. Powder X-ray diffraction data for the bulk sample that contains 2($\mathbf{C_6H_3Cl_4N}$)·(**Azo**) (blue) along with the theoretical pattern for 2($\mathbf{C_6H_3Cl_4N}$)·(**Azo**) (orange).

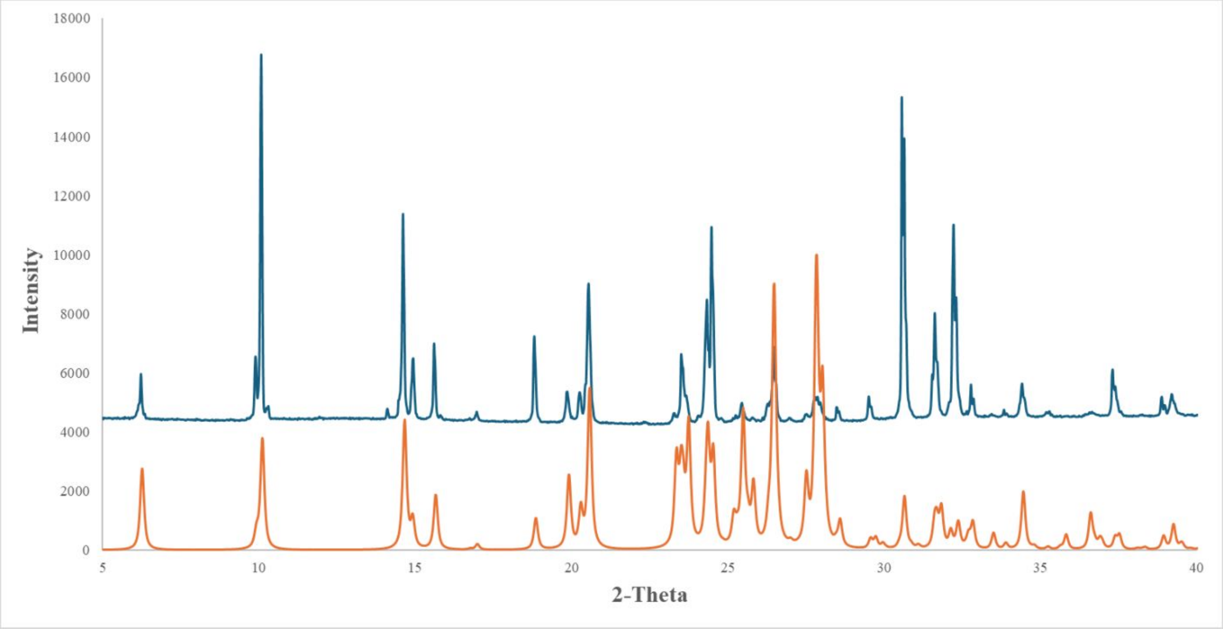

Figure S8. Powder X-ray diffraction data for the bulk sample that contains 2($\mathbf{C_6H_4Cl_3N}$)·(**BPE**) (blue) along with the theoretical pattern for 2($\mathbf{C_6H_4Cl_3N}$)·(**BPE**) (orange).

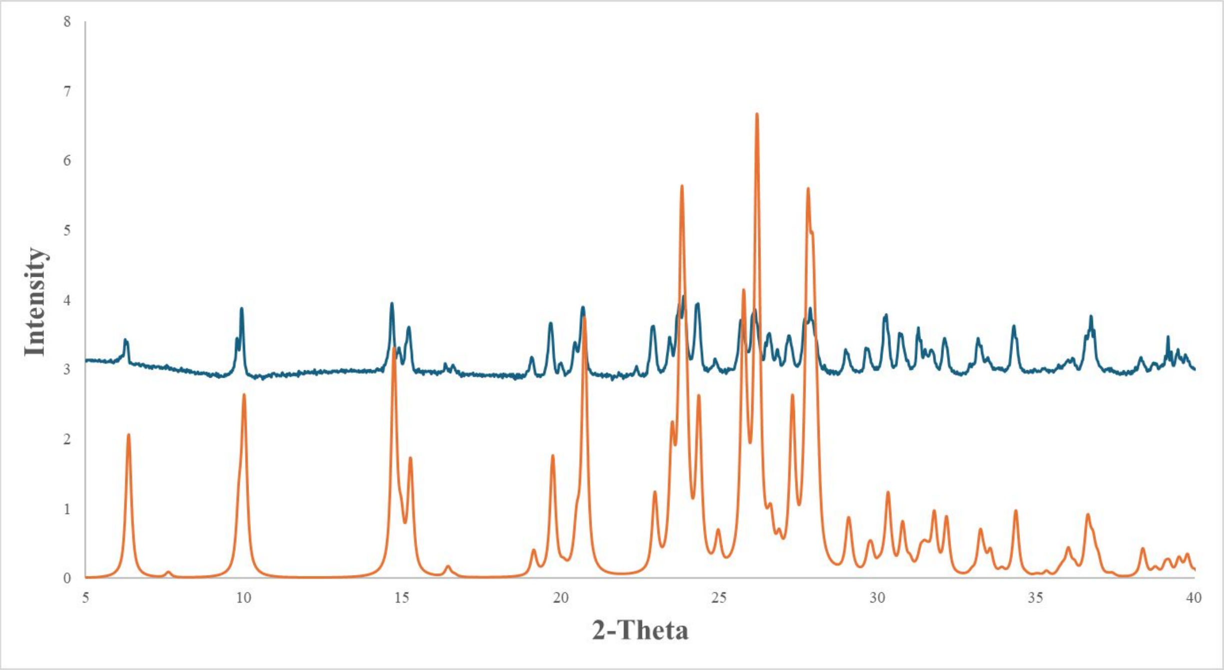

Figure S9. Powder X-ray diffraction data for the bulk sample that contains 2(**$C_6H_4Cl_3N$**)·(**BPA**) (blue) along with the theoretical pattern for 2(**$C_6H_4Cl_3N$**)·(**BPA**) (orange).

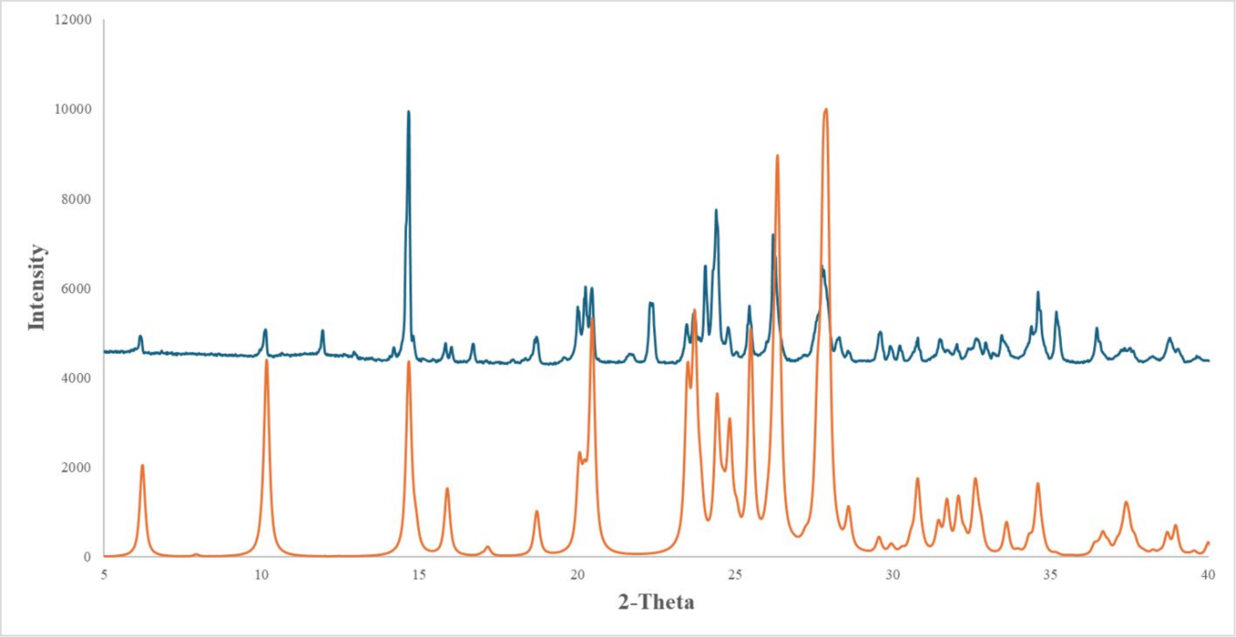

Figure S10. Powder X-ray diffraction data for the bulk sample that contains 2(**$C_6H_4Cl_3N$**)·(**Azo**) (blue) along with the theoretical pattern for 2(**$C_6H_4Cl_3N$**)·(**Azo**) (orange).

4. Density Functional Theory Calculation Figures

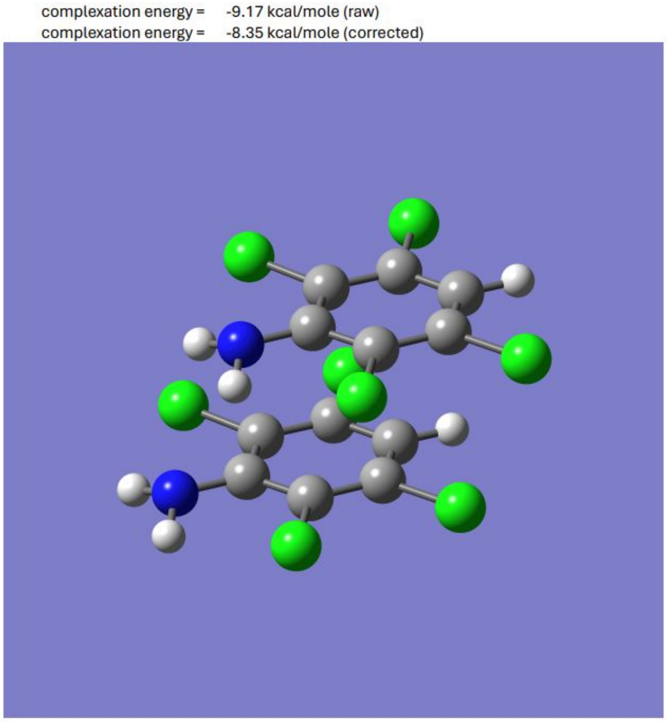

Figure S11. View of the orientation and binding energies of the observed homogenous and face-to-face π-π stacking pattern of $\mathbf{C_6H_3Cl_4N}$.

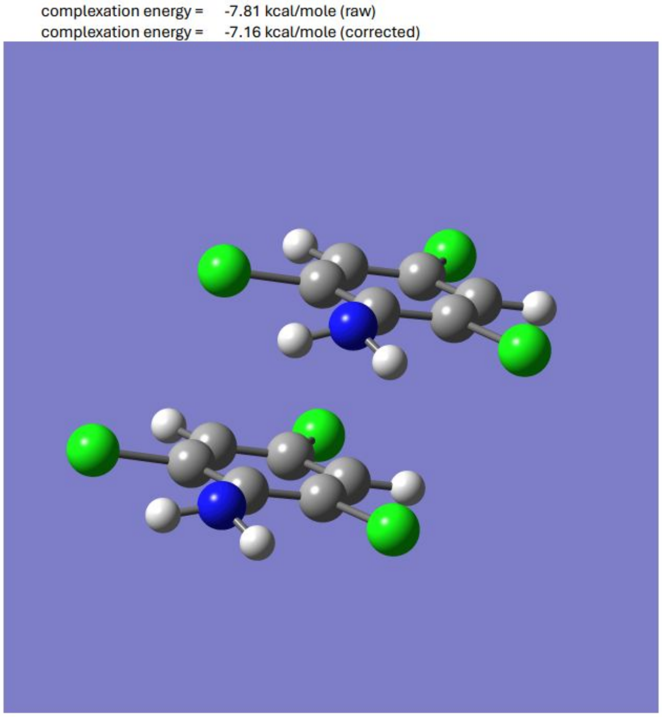

Figure S12. View of the orientation and binding energies of the observed homogenous and face-to-face π-π stacking pattern of $\mathbf{C_6H_4Cl_3N}$.

complexation energy = -4.22 kcal/mole (raw)
complexation energy = -3.77 kcal/mole (corrected)

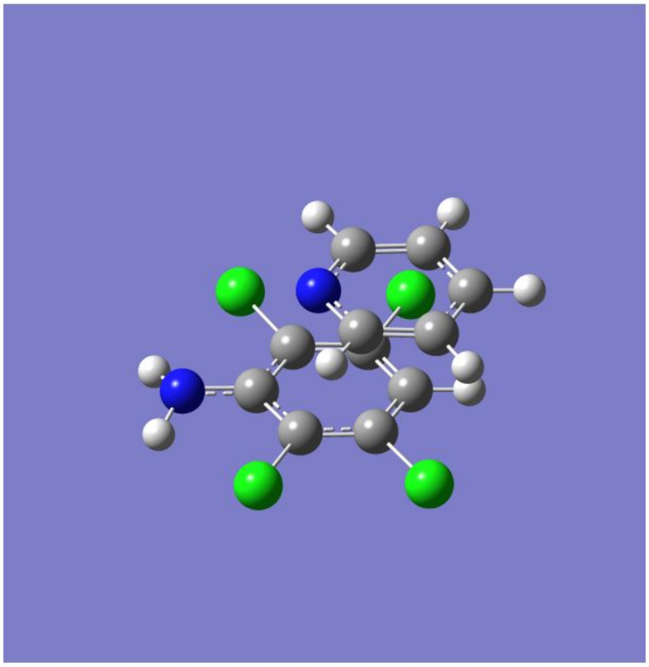

Figure S13. View of the orientation and binding energies of the theoretical heterogeneous and face-to-face π-π stacking pattern of **$C_6H_3Cl_4N$** and a mock pyridine in a *syn* orientation.

complexation energy = -4.82 kcal/mole (raw)
complexation energy = -4.37 kcal/mole (corrected)

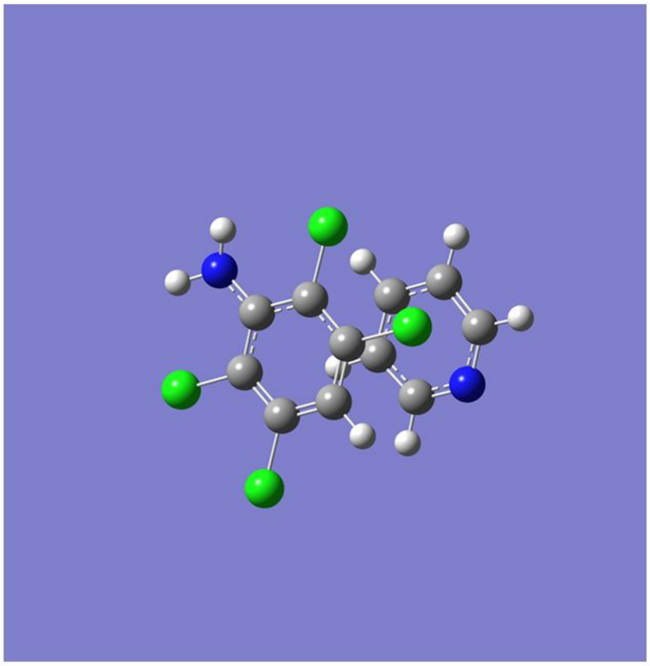

Figure S14. View of the orientation and binding energies of the theoretical heterogeneous and face-to-face π-π stacking pattern of **$C_6H_3Cl_4N$** and a mock pyridine in an *anti* orientation.

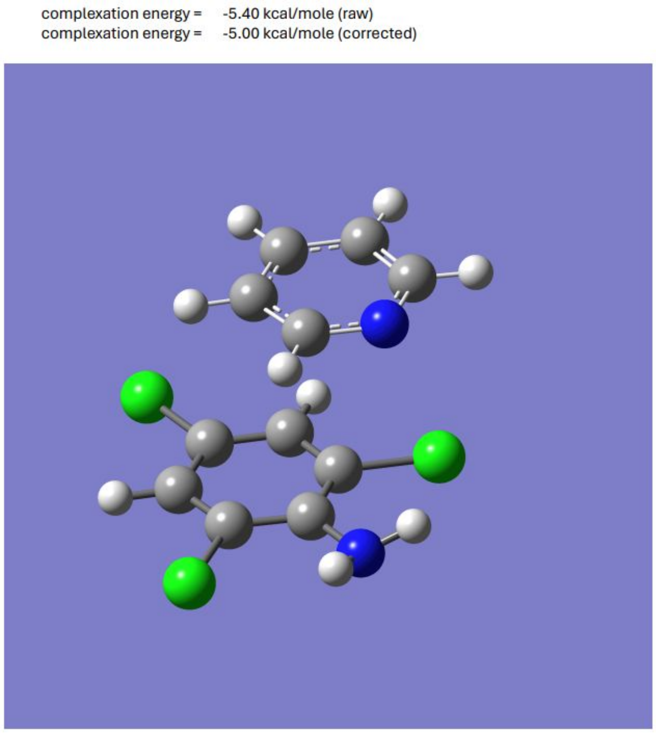

Figure S15. View of the orientation and binding energies of the theoretical heterogeneous and face-to-face π-π stacking pattern of $\mathbf{C_6H_4Cl_3N}$ and a mock pyridine in a *syn* orientation.

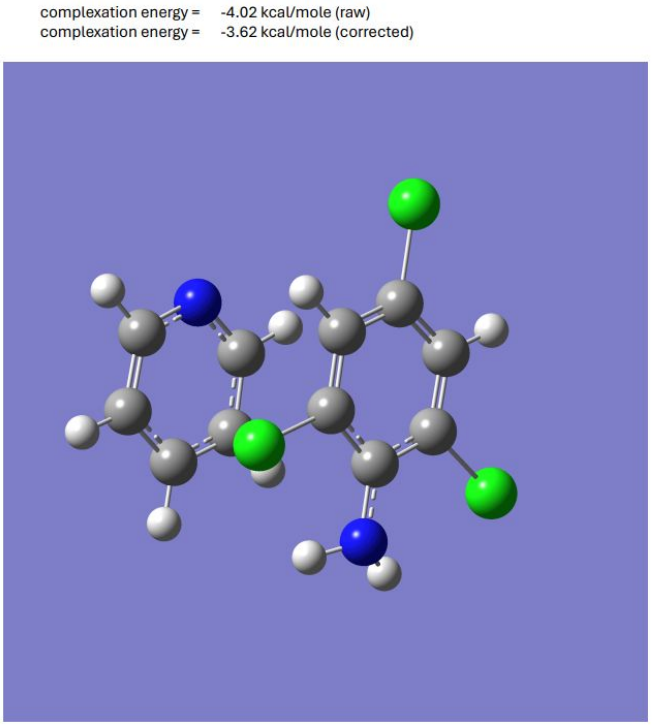

Figure S16. View of the orientation and binding energies of the theoretical heterogeneous and face-to-face π-π stacking pattern of $\mathbf{C_6H_4Cl_3N}$ and a mock pyridine in an *anti* orientation.

5. Solution ^{1}H NMR Spectroscopic Data

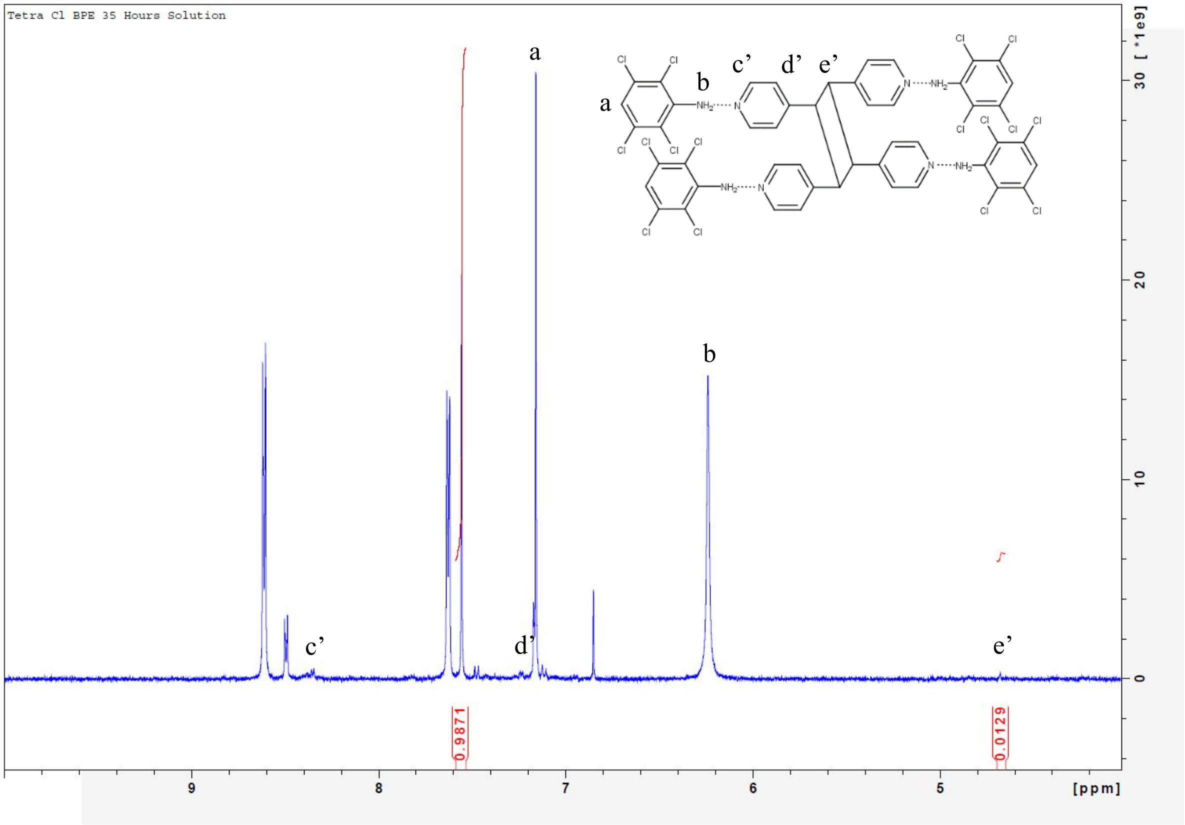

Figure S17: ^{1}H NMR spectrum of the solution containing $\mathbf{C_6H_4Cl_3N}$ and **BPE** after 35 hours of UV irradiation reaching a yield of 1.3% for the [2 + 2] cycloaddition reaction (400 MHz, DMSO-d_6).

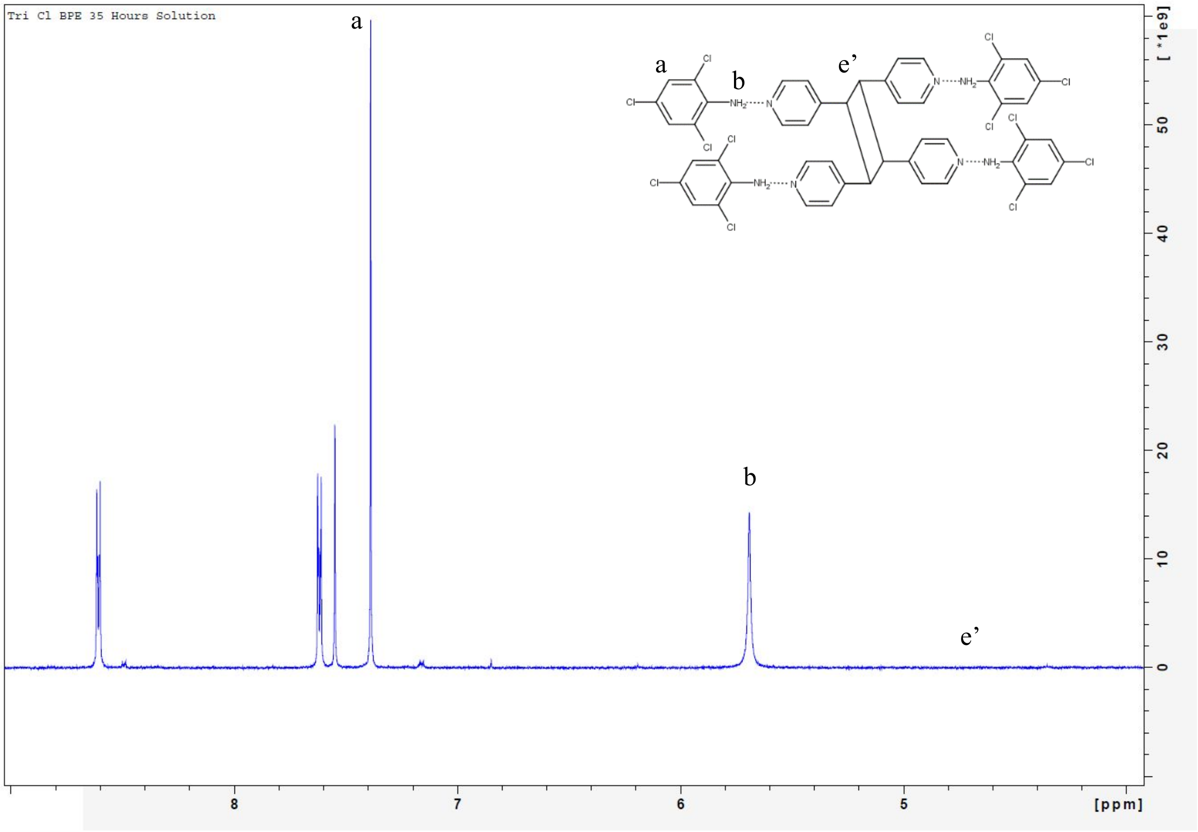

Figure S18: ^{1}H NMR spectrum of the solution containing **$C_6H_3Cl_4N$** and **BPE** after 35 hours of UV irradiation confirming no [2 + 2] cycloaddition reaction occurred (400 MHz, DMSO-d_6).

References

1. Bruker (2021). *APEX6*, *SAINT* and *SADABS*. Bruker AXS Inc., Madison, Wisconsin, USA.

2. G. M. Sheldrick, *Acta Crystallogr.*, 2015, A**71**, 3-8.

3. G. M. Sheldrick, *Acta Crystallogr.*, 2015, C**71**, 3-8.

4. O. V. Dolomanov, L. J. Bourhis, R. J. Gildea, J. A. K. Howard, H. Puschmann, *J. Appl. Cryst.*, 2009, **42**, 339-341.
